# LINC02532 Contributes to Radiosensitivity in Clear Cell Renal Cell Carcinoma through the miR-654-5p/YY1 Axis

**DOI:** 10.3390/molecules26227040

**Published:** 2021-11-22

**Authors:** Xiaoguang Zhou, Bowen Zeng, Yansheng Li, Haozhou Wang, Xiaodong Zhang

**Affiliations:** 1 Department of Urology, Beijing Chaoyang Hospital, Capital Medical University, Beijing 100020, China; Xiaoguang.zhou@hotmail.com (X.Z.); drzeng0124@163.com (B.Z.); liyansheng128@163.com (Y.L.); wanghzmail@sina.cn (H.W.); 2 Department of Urology, Affiliated Hospital of Sergeant School of Army Medical University, Shijiazhuang 050044, China

**Keywords:** clear cell renal cell carcinoma, radioresistance, LINC02532, miR-654-5p, YY1

## Abstract

Background: Studies have shown that long non-coding RNAs (lncRNAs) play essential roles in tumor progression and can affect the response to radiotherapy, including in clear cell renal cell carcinoma (ccRCC). LINC02532 has been found to be upregulated in ccRCC. However, not much is known about this lncRNA. Hence, this study aimed to investigate the role of LINC02532 in ccRCC, especially in terms of radioresistance. Methods: Quantitative real-time PCR was used to detect the expression of LINC02532, miR-654-5p, and YY1 in ccRCC cells. Protein levels of YY1, cleaved PARP, and cleaved-Caspase-3 were detected by Western blotting. Cell survival fractions, viability, and apoptosis were determined by clonogenic survival assays, CCK-8 assays, and flow cytometry, respectively. The interplay among LINC02532, miR-654-5p, and YY1 was detected by chromatin immunoprecipitation and dual-luciferase reporter assays. In addition, in vivo xenograft models were established to investigate the effect of LINC02532 on ccRCC radioresistance in 10 nude mice. Results: LINC02532 was highly expressed in ccRCC cells and was upregulated in the cells after irradiation. Moreover, LINC02532 knockdown enhanced cell radiosensitivity both in vitro and in vivo. Furthermore, YY1 activated LINC02532 in ccRCC cells, and LINC02532 acted as a competing endogenous RNA that sponged miR-654-5p to regulate YY1 expression. Rescue experiments indicated that miR-654-5p overexpression or YY1 inhibition recovered ccRCC cell functions that had been previously impaired by LINC02532 overexpression. Conclusions: Our results revealed a positive feedback loop of LINC02532/miR-654-5p/YY1 in regulating the radiosensitivity of ccRCC, suggesting that LINC02532 might be a potential target for ccRCC radiotherapy. This study could serve as a foundation for further research on the role of LINC02532 in ccRCC and other cancers.

## 1. Background

Renal cell carcinoma (RCC) is a serious threat to human health, affecting 30 million people worldwide each year and causing more than 100,000 deaths annually [1]. Clear cell renal cell carcinoma (ccRCC), the most common subtype of RCC, accounts for 80–90% of RCC cases, and has a higher infiltration capacity and recurrence rate than other RCC subtypes [2]. Unfortunately, due to its acquired resistance to chemotherapy and radiotherapy, the 5-year survival rate of patients with ccRCC is lower than 20% [3,4]. Therefore, it is important to explore effective and alternative approaches to enhance ccRCC sensitivity to chemotherapy or radiotherapy.

Radiotherapy is a common treatment for cancer [5] since ionizing radiation (IR) damages cancer cells by inducing DNA damage, such as DNA double-strand breaks (DSBs) [6]. Unfortunately, the response of cancer cells to DNA damage enhances the repair of DNA DSBs, and results in acquired resistance to IR [7]. Over the past decades, researchers have gradually uncovered the cellular signaling pathways for DNA break repair [8,9]. The roles and mechanisms of non-coding RNAs (ncRNAs) in this process have attracted attention [10,11].

Long non-coding RNAs (lncRNAs) are ncRNAs that are longer than 200 nucleotides (nt) in length. Recent studies have reported the pivotal roles of lncRNAs in regulating cancer resistance to radiotherapy [12,13,14]. For example, in colorectal cancer, the lncRNA HOTAIR is upregulated and promotes radioresistance by regulating autophagy [15]. Similarly, in nasopharyngeal carcinoma, the lncRNA MINCR regulates irradiation resistance by activating the AKT/PI3K axis [16]. Long intergenic noncoding RNA 2532 (LINC02532) is a lncRNA that has been rarely reported, with only one previous study by Zhang et al. reporting its oncogenic role in gastric cancer [17]. Based on early research and data from The Cancer Genome Atlas (TCGA), we found that LINC02532 was upregulated in ccRCC. Hence, we further investigated whether LINC02532 is involved in the development of radioresistance in ccRCC.

MicroRNAs (miRNAs) are another type of ncRNA that are generally 20–22 nt in length. By binding to the 3′ untranslated region (3′ UTR) of specific mRNAs, miRNAs can degrade the target mRNA or repress its transcription [18]. Dysregulation of miRNAs has been associated with various biological processes [19,20,21,22], including the poor response of cancer cells to radiotherapy [23]. MiR-218-5p enhances the radiosensitivity of lung carcinoma cells by inhibiting PRKDC activity [24]. Moreover, miR-181a reduces the radiosensitivity of non-small-cell lung cancer by regulating PTEN [25]. MiR-654-5p has been reported to play an important role in cancer development [26,27,28,29]. However, whether it is related to radioresistance in ccRCC remains unclear and is yet to be investigated.

Yin Yang 1 (YY1), a GLI-Krüppel zinc finger protein, is a DNA/RNA-binding transcription factor important in tumorigenesis and cancer progression [30,31,32,33]. In fact, by binding to the promoter region of lncRNA PVT1, YY1 promotes the progression of lung cancer [34]. Moreover, the lncRNA Sox2ot represses Sox2 expression by interacting with YY1, thereby promoting cortical neurogenesis [35]. Aside from those roles, YY1 is also associated with radioresistance in cancer [36,37]. Therefore, we hypothesized that YY1 might participate in LINC02532-mediated radioresistance in ccRCC.

In this study, we found that LINC02532 is involved in regulating radioresistance in ccRCC. The knockdown of LINC02532 accelerated the sensitivity of ccRCC cells to irradiation both in vitro and in vivo. Mechanistically, LINC02532 contributed to radioresistance by sponging miR-654-5p to regulate YY1 expression. Furthermore, YY1 could transcriptionally activate LINC02532 in ccRCC cells. In conclusion, this study found a “LINC02532–miR-654-5p–YY1” loop in ccRCC and suggested that LINC02532 might be a potential therapeutic target for radiotherapy in ccRCC.

## 2. Methods

### 2.1. Cell Lines and Cell Culture

HK-2, 786-O, A-498, and Caki-1 cell lines were obtained from the American Type Culture Collection (ATCC, Manassas, VA, USA). HK-2 and A-498 cells were grown in DMEM (Gibco, Los Angeles, CA, USA) supplemented with 10% fetal bovine serum (FBS; Invitrogen, Carlsbad, CA, USA), whereas 786-O cells were grown in RPMI-1640 medium (Gibco) supplemented with 10% FBS, and Caki-1 cells were grown in McCoy’s 5A medium (Thermo Fisher Scientific, Waltham, MA, USA) that was also supplemented with 10% FBS. All cells were maintained at 37 °C in an incubator in a 5% CO_2_ atmosphere.

### 2.2. Cell Transfection

Specific small interfering RNAs (siRNAs) targeting LINC02532 (si-LINC02532#1, si-LINC02532#2, and si-LINC02532#3), and YY1 (si-YY1), the siRNA control (si-NC), as well as miR-654-5p mimics (miR-mimics), mimic control (miR-NC), a LINC02532 overexpression vector (LINC02532), and an empty overexpression vector (vector) were obtained from RiboBio (Guangzhou, China). The sh-LINC02532 lentivirus and its control lentiviruses were obtained from GenePharma (Shanghai, China).

Transient transfections were performed using Lipofectamine 2000 (Invitrogen, Carlsbad, CA, USA) following the manufacturer’s protocol. In brief, 786-O and A-498 cells (4 × 10^5^ cells/well) were seeded in a 6-well plate. When the cells were 60–80% confluence, the mixed solution of target plasmids and Lipofectamine 2000 (dilute 4 μg target plasmids and 10 μL Lipofectamine 2000 with 250 μL Opti-MEM medium (Gibco), respectively, then mix the diluents together and set it for 20 min) were added to each well. Six hours later, the culture medium was renewed for further culture. The cells were harvested at 48 h after transfection.

Lentiviral infection was used to generate the stable 786-O cell. In brief, 786-O cells were seeded in a 24-well plate (1 × 10^5^ cells/well) and grown to 40–50% confluence. Then, the 786-O cells were infected with control lentiviruses and sh-LINC02532 lentiviruses (the multiplicity of infection for 786-O cells is 20) with 4 μg/mL of polybrene (Sigma-Aldrich, St. Louis, MO, USA). After infection, the stable 786-O cells were selected by treating with puromycin (2 μg/mL, Sigma-Aldrich) for 7 days.

### 2.3. Quantitative Real-Time PCR (qRT-PCR)

Total RNA was extracted from the samples using TRIzol reagent (Invitrogen). Then, cDNA was synthesized using the One-Step PrimeScript RT-PCR Kit (Takara Bio, Shiga, Japan) or the miScript II RT Kit (QIAGEN, Hilden, Germany). qRT-PCR was carried out on an ABI 7500 qPCR instrument (Applied Biosystems, Foster City, CA, USA) using the SYBR Green PCR kit (Qiagen). The relative expression was normalized to that of *GAPDH* or U6 using the 2^−ΔΔC^^T^ method. Primer sequences are listed in Table S1.

### 2.4. Subcellular Fractionation

The subcellular fractions were collected using the NE-PER™ Nuclear and Cytoplasmic Extraction Kit (Thermo Fisher). In brief, the nuclear and cytoplasmic fractions of the 786-O and A-498 cells were isolated according to the manufacturer’s protocol. Then, the expression ratios of LINC02532 in the nuclear and cytoplasmic fractions were detected by qRT-PCR, with U6 as the nuclear control, and *GAPDH* as the cytoplasmic control.

### 2.5. CCK-8 Assay

Cell viability was assessed using the cell counting kit-8 (CCK-8, Dojindo, Tokyo, Japan) assay. The cells were seeded in 96-well plates for 24 h before IR (4 Gy) treatment, followed by incubation with 10 μL of CCK-8 solution for 2 h. Absorbance values were then measured at a wavelength of 450 nm.

### 2.6. Radiation Treatment

Cells were seeded in six-well plates and exposed to IR with gradient doses on the order of 0–8 Gy. Two weeks later, the colonies were fixed and stained for 15 min with 0.1% crystal violet solution (Sigma-Aldrich). The number of colonies was counted, and the survival fraction was calculated.

### 2.7. Cell Apoptosis Detection

Cells were seeded in six-well plates and incubated for 24 h before IR (4 Gy) treatment. The percentage of apoptotic cells was evaluated using a Dead Cell Apoptosis Kit with Annexin V FITC and PI (Invitrogen), following the manufacturer’s instructions.

### 2.8. Immunofluorescence Staining

Cells were cultured on glass coverslips. Then, 1 or 4 h after irradiation, the coverslips were fixed in 4% paraformaldehyde and permeabilized with 0.5% triton X-100. Afterwards, the cells were incubated with anti-γ-H2AX (ab26350; Abcam, Cambridge, MA, USA) at 4 °C overnight, followed by incubation with secondary antibody for 1 h at 37 °C, and then staining with 4′,6-diamidino-2-phenylindole (DAPI). Finally, the cells were observed under a fluorescence microscope (Olympus, Tokyo, Japan).

### 2.9. Fluorescence In Situ Hybridization (FISH)

LINC02532 probes were obtained from RiboBio (Guangzhou, China). After cell fixation and permeabilization, the cells were hybridized with the probe at 37 °C overnight. Subsequently, the slides were stained with DAPI, and images were taken under a fluorescence microscope (Olympus, Tokyo, Japan).

### 2.10. Western Blotting

Proteins were extracted from the cells using radioimmunoprecipitation assay buffer (Invitrogen), and then, the sample concentration was assessed using a bicinchoninic acid protein quantification kit (Thermo Fisher Scientific). Equal amounts of proteins were separated by 10% sodium dodecyl sulfate polyacrylamide gel electrophoresis (Bio-Rad, Hercules, CA, USA), transferred to polyvinylidene fluoride membranes (Millipore, Temecula, CA), blocked with 5% skim milk, and then incubated with the following primary antibodies: anti-YY1 (#46395, Cell Signaling Technology, Danvers, MA, USA, 1:1000), anti-PARP (#9542, Cell Signaling Technology, 1:1000), anti-Caspase-3 (#9662, Cell Signaling Technology, 1:1000), and anti-GAPDH (#5174, Cell Signaling Technology, 1:1000). Next, the membranes were incubated with secondary antibodies. Protein signals were observed using enhanced chemiluminescence (Bio-Rad), with GAPDH as an internal control (Olympus, Tokyo, Japan).

### 2.11. Luciferase Reporter Assay

Luciferase reporter vectors containing the LINC02532 wild-type, YY1 wild-type, and respective mutation sequences, as well as the YY1 target sequence within the LINC02532 promoter, were obtained from GenePharma (Shanghai, China), and 786-O and A-498 cells were seeded into 24-well plates and then co-transfected with the reporter plasmid and miRNA mimics. At 48 h post-transfection, luciferase activity was measured using a Dual-Luciferase Reporter Assay System (Promega, Madison, WI, USA).

### 2.12. Chromatin Immunoprecipitation (ChIP)

A commercially available kit (Beyotime, Jiangsu, China) was used for the ChIP assay. Briefly, cells were treated with 1% formaldehyde for crosslinking and then sonicated on ice. Anti-YY1 (#46395, Cell Signaling Technology) or anti-IgG (#2729, Cell Signaling Technology) antibodies were added to the stained chromatin and incubated overnight. The precipitated chromatin DNA was recovered and analyzed by qRT-PCR. The primer sequences are shown in Table S2.

### 2.13. Xenograft Assay

Ten male nude mice (6 weeks old) purchased from the Chinese Academy of Medical Sciences (Beijing, China) were maintained in a pathogen-free environment and allowed free access to water and food. A total of 5 × 10^6^ 786-O cells infected with control lentiviruses or sh-LINC02532 lentiviruses were subcutaneously injected into the mice (*n* = 5 per group). At day 10 after cell injection, the mice were exposed to 10 Gy IR once. Changes in tumor volume were measured every 5 d for 30 days and were calculated using the following formula: volume (mm^3^) = (length × width^2^)/2. At 30 d post-injection, the animals were euthanized by cervical dislocation after anesthesia, and then, the tumors were resected and weighed. LINC02532 and miR-654-5p expression in the xenograft tumors were determined by qRT-PCR. Protein expression of YY1 was determined by Western blot. Animal experiments were approved by the Animal Care and Use Committee of Beijing Chaoyang Hospital (Number: 2020-541) and were performed in accordance with the NIH Animal Care Guidelines.

### 2.14. Statistical Analysis

All experiments were conducted three times independently, and the data are shown as the mean ± standard deviation. Statistical analyses were performed using the GraphPad Prism 7.0 (San Diego, CA, USA) using a Student’s *t*-test or one-way analysis of variance. Statistical significance was set at *p* < 0.05.

## 3. Results

### 3.1. Knockdown of LINC02532 Suppresses Cell Viability in ccRCC Cells

**Figure 1:**
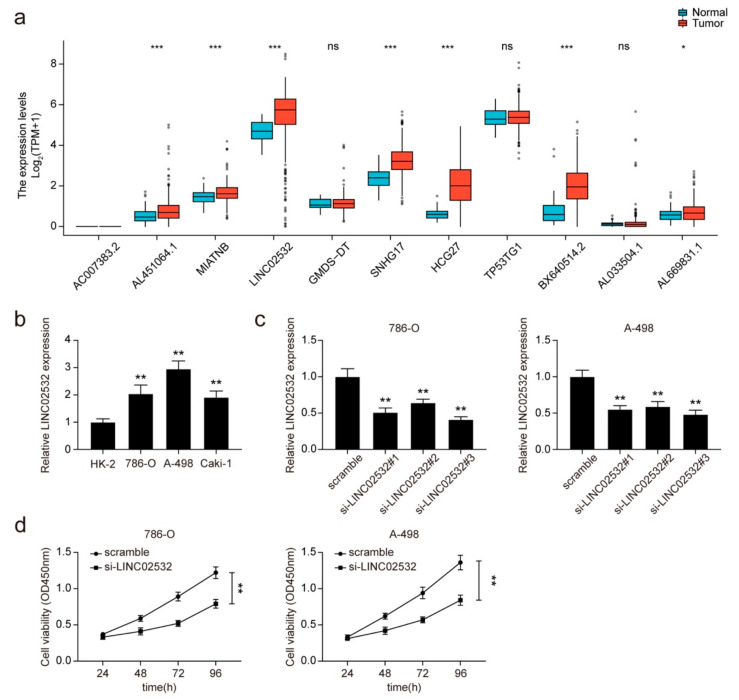
LINC02532 is highly expressed in clear cell renal cell carcinoma (ccRCC). (**a**) The expression of lncRNA AC007383.2, AL451064.1, MIATNB, LINC02532, GMDS-DT, SNHG17, HCG27, TP53TG1, BX640514.2, AL033504.1, and AL669831.1 in The Cancer Genome Atlas (TCGA) kidney renal clear cell carcinoma cohort. (**b**) qRT-PCR detection of LINC02532 expression in ccRCC cell lines. (**c**) qRT-PCR detection of LINC02532 expression in 786-O and A-498 cells transfected with LINC02532 small interfering RNAs (siRNAs). (**d**) Cell viability was monitored in 786-O and A-498 cells transfected with si-LINC02532 by cell counting kit-8 (CCK-8) assays. * *p* < 0.05, ** *p* < 0.01, *** *p* < 0.001.

First, 19 differentially expressed lncRNAs were identified in the primary ccRCC cells (Table S3). Among them, 11 lncRNAs showed a higher expression in ccRCC, including AC007383.2, AL451064.1, MIATNB, LINC02532, GMDS-DT, SNHG17, HCG27, TP53TG1, BX640514.2, AL033504.1, and AL669831.1. Next, we evaluated the expression of these 11 lncRNAs in TCGA kidney renal clear cell carcinoma cohort. LINC02532 showed the highest expression in tumor samples and was selected for further functional assays (Figure 1a). Likewise, qRT-PCR results revealed that LINC02532 was highly expressed in ccRCC cells (Figure 1b). Considering the higher expression of LINC02532 in 786-O and A-498 cells, they were used for the subsequent experiments.

Three siRNAs targeting LINC02532 were synthesized and transfected into 786-O and A-498 cells to knock down LINC02532 expression. As a result, all siRNAs silenced LINC02532 expression in 786-O and A-498 cells (Figure 1c), and si-LINC02532#3 was used for the subsequent loss-of-function experiments because of its knockdown efficiency. Subsequent CCK-8 results showed that LINC02532 knockdown led to a remarkable decrease in the viability of 786-O and A-498 cells (Figure 1d). These results indicate that LINC02532 was highly expressed in ccRCC, and knockdown of LINC02532 inhibited cell viability in ccRCC cells.

### 3.2. LINC02532 Knockdown Accelerates Radiosensitivity in ccRCC

**Figure 2:**
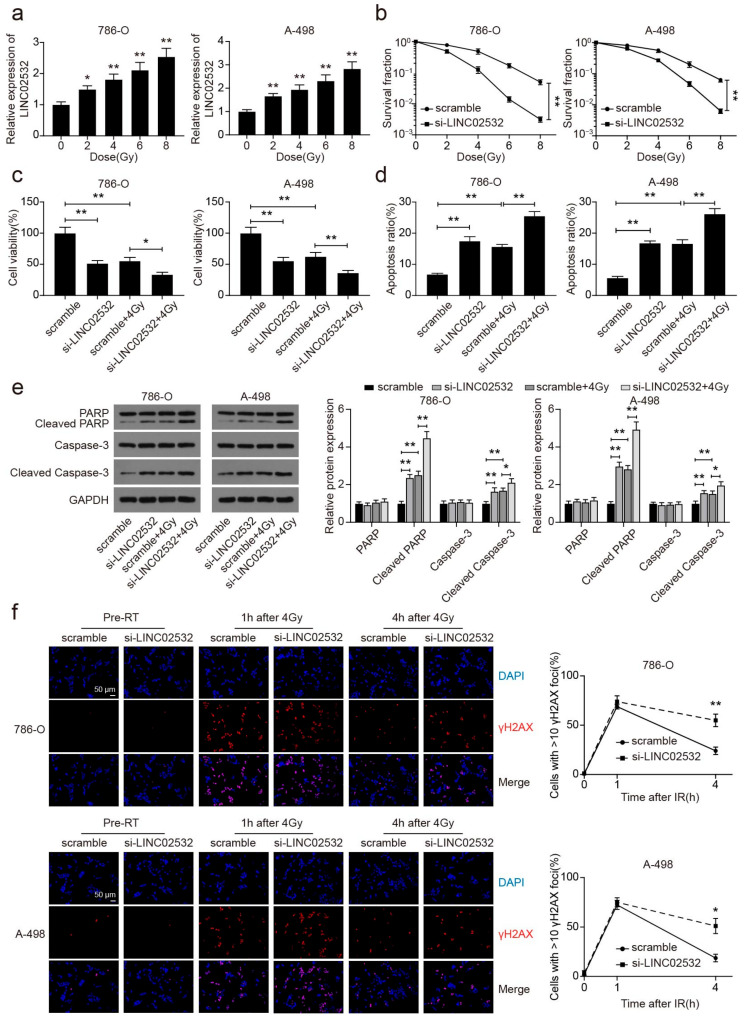
LINC02532 knockdown potentiates radiosensitivity of clear cell renal cell carcinoma (ccRCC) cells. (**a**) qRT-PCR detection of LINC02532 expression in 786-O and A-498 cells under different irradiation exposures. (**b**) The surviving fraction of 786-O and A-498 cells after transfections. (**c**) Effect of LINC02532 inhibition on the viability of ccRCC cells under ionizing radiation (IR) treatment. (**d**) Effect of LINC02532 inhibition on the apoptosis of ccRCC cells under IR treatment. (**e**) Western blotting detection of PARP, cleaved-PARP, Caspase-3, and cleaved-Caspase-3 protein expression. (**f**) Immunofluorescence staining of γ-H2AX in ccRCC cells (magnification, ×400, scale = 50 μm). * *p* < 0.05, ** *p* < 0.01.

Based on the previous results, we wondered whether LINC02532 is correlated with radioresistance in ccRCC. As shown in Figure 2a, IR treatment induced a dose-dependent increase in LINC02532 expression in 786-O and A-498 cells. Subsequent radiation clonogenic survival assays showed that LINC02532 knockdown enhanced the sensitivity of 786-O and A-498 cells to radiation (Figure 2b). In addition, knockdown of LINC02532 reduced cell viability (Figure 2c), promoted apoptosis (Figure 2d, Figure S1), and increased the levels of cleaved PARP and cleaved-Caspase-3 (Figure 2e) in IR-treated 786-O and A-498 cells. Furthermore, immunofluorescence staining of γ-H2AX showed that γ-H2AX foci resolution at 4 h after 4 Gy irradiation was remarkably delayed in 786-O and A-498 cells transfected with si-LINC02532 (Figure 2f), indicating that LINC02532 influenced ccRCC radiosensitivity by affecting the repair of DNA DSBs. Taken together, our results suggest that knockdown of LINC02532 potentiates the radiosensitivity of ccRCC cells by delaying DNA DSB repair.

### 3.3. YY1 Transcriptionally Activates LINC02532 in ccRCC Cells

**Figure 3:**
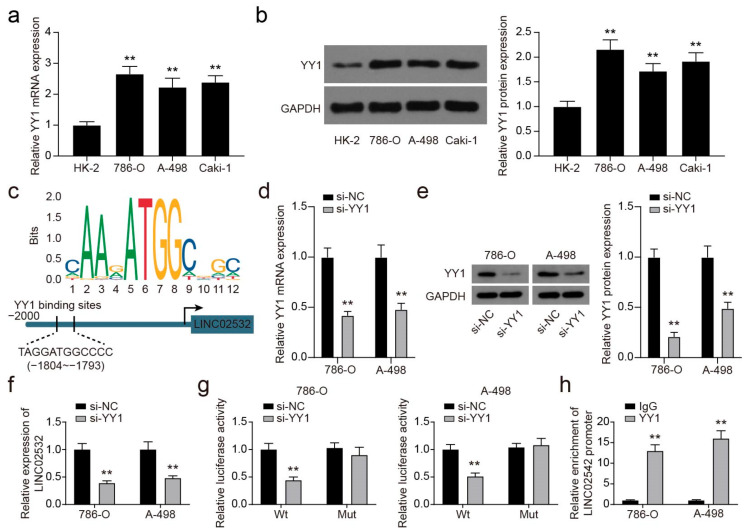
YY1 transcriptionally activates LINC02532 in clear cell renal cell carcinoma (ccRCC) cells. (**a**,**b**) The mRNA and protein expression of YY1 in ccRCC cells. (**c**) YY1 binding site in the LINC02532 promoter. (**d**,**e**) The mRNA and protein expression of YY1 in ccRCC cells transfected with si-YY1. (**f**) LINC02532 expression in cells transfected with si-YY1. (**g**) Binding relationship between YY1 and LINC02532 promoter was confirmed by luciferase reporter assays. (**h**) qRT-PCR detection of the chromatin immunoprecipitation (ChIP) products confirmed the interaction between YY1 and the LINC02532 promoter. ** *p* < 0.01.

Previous studies have reported that the YY1 transcription factor can transcriptionally activate various lncRNAs [34,38,39,40,41]. Hence, we investigated whether YY1 could regulate LINC02532 expression at the transcriptional level. Through qRT-PCR and Western blotting, we found that the mRNA and protein expression of YY1 was higher in ccRCC cells (Figure 3a,b). Next, using the JASPAR webtool, the YY1 binding site on the LINC02532 promoter was predicted and is shown in Figure 3c.

To explore whether LINC02532 is a downstream target of YY1, YY1 was knocked down by siRNA in 786-O and A-498 cells (Figure 3d,e), and this led to a significant decrease in LINC02532 expression (Figure 3f). Subsequent luciferase reporter assays showed a decrease in luciferase activity after YY1 inhibition in the wild-type group, whereas that in the mutant group did not change after transfection (Figure 3g). Moreover, ChIP assay results showed that the LINC02532 promoter was specifically pulled down by a YY1-specific antibody but not by the control antibody (Figure 3h), suggesting that YY1 binds the LINC02532 promoter. Taken together, these findings suggest that YY1 could transcriptionally activate LINC02532 expression in ccRCC cells.

### 3.4. LINC02532 Sponges miR-654-5p to Regulate YY1 Expression in ccRCC Cells

**Figure 4:**
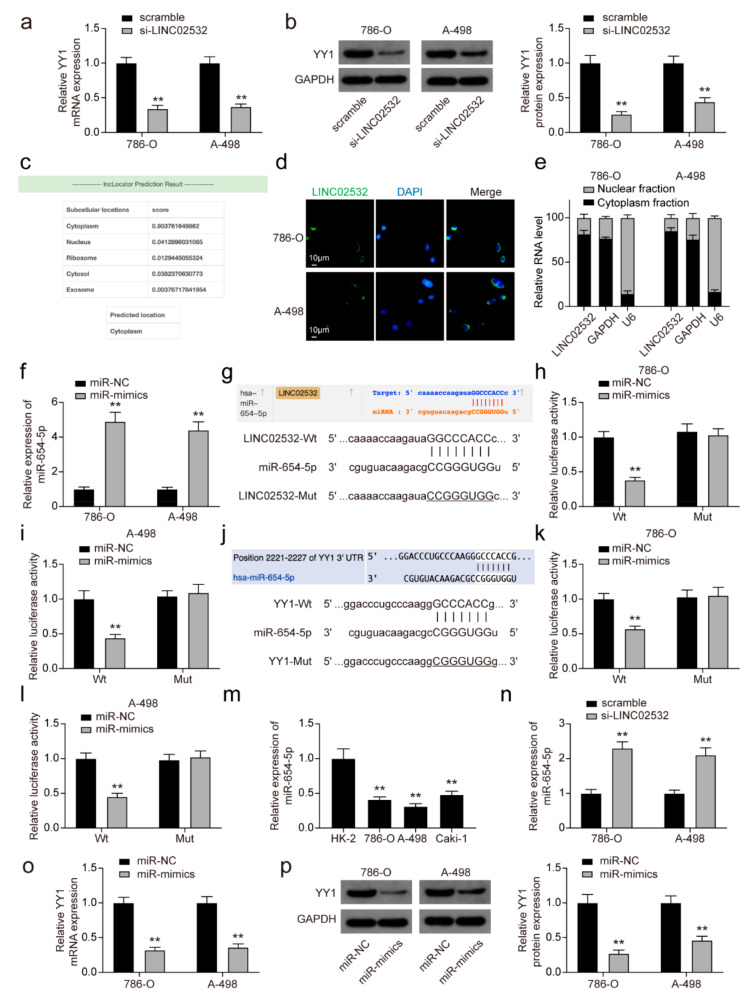
LINC02532 regulates YY1 in clear cell renal cell carcinoma (ccRCC) cells by sponging miR-654-5p. (**a**,**b**) The mRNA and protein expression of YY1 in ccRCC cells transfected with si-LINC02532. (**c**) LINC02532 was predicted to be localized in cytoplasm by the lncLocator webtool (http://www.csbio.sjtu.edu.cn/bioinf/lncLocator/, accessed on 11 July 2020). (**d**) FISH results showed that LINC02532 was localized in the cytoplasm. LINC02532 probes were stained green. Nuclei were stained blue (scale = 10 μm). (**e**) The specific distribution of LINC02532 in 786-O and A-498 cells. (**f**) qRT-PCR detection of the transfection efficiency of miR-654-5p mimics. (**g**) Predicted complementary sites between LINC02532 and miR-654-5p. (**h**,**i**) Luciferase activities of LINC02532-Wt and LINC02532-Mut in 786-O and A-498 cells transfected with miR-654-5p mimic or mimics NC. (**j**) Predicted complementary sites between miR-654-5p and YY1. (**k**,**l**) Luciferase activities of YY1-Wt and YY1-Mut in 786-O and A-498 cells transfected with miR-654-5p mimic or mimics NC. (**m**) qRT-PCR detection of miR-654-5p expressions in ccRCC cells. (**n**) The expression of miR-654-5p in 786-O and A-498 cells transfected with si-LINC02532. (**o**,**p**) YY1 mRNA and protein levels in 786-O and A-498 cells transfected with miR-mimics. ** *p* < 0.01.

The aforementioned findings indicated that LINC02532 is a target of YY1, and thus, we investigated whether LINC02532 could regulate YY1. qRT-PCR and Western blotting results showed that YY1 expression was suppressed by the knockdown of LINC02532 (Figure 4a,b), indicating the regulatory effect of LINC02532 on YY1. As the function of lncRNA depends on its subcellular localization [42], we explored the distribution of LINC02532 in ccRCC cells. Using the lncLocator webtool, we determined that LINC02532 in ccRCC was mainly located in the cytoplasm (Figure 4c). Additionally, subcellular fraction and FISH assays confirmed this cytoplasmic location of LINC02532 (Figure 4d,e). Given that cytoplasmic lncRNAs function as competing endogenous RNAs in cancer [43,44], we speculated that LINC02532 would regulate YY1 expression in this manner.

Using the starBase and TargetScan databases, miR-654-5p was found to bind both LINC02532 and YY1. Transfection efficiency analysis showed that miR-654-5p mimics significantly upregulated miR-654-5p expression in 786-O and A-498 cells (Figure 4f). Subsequent luciferase reporter assays revealed reduced luciferase activity in the LINC02532-Wt and YY1-Wt groups after transfection with miR-mimics; however, no significant changes in luciferase activity were found in the mutant groups after transfection (Figure 4g–l). In addition, miR-654-5p expression was significantly downregulated in ccRCC cells (Figure 4m). In contrast, LINC02532 knockdown promoted miR-654-5p expression in 786-O and A-498 cells (Figure 4n). Furthermore, decreased mRNA and protein levels of YY1 were observed when miR-654-5p was overexpressed (Figure 4o,p). Overall, these results indicate that LINC02532 upregulates YY1 expression by sponging miR-654-5p.

### 3.5. miR-654-5p Overexpression Restores the Effect of LINC02532 Overexpression on Radiosensitivity in ccRCC Cells

**Figure 5:**
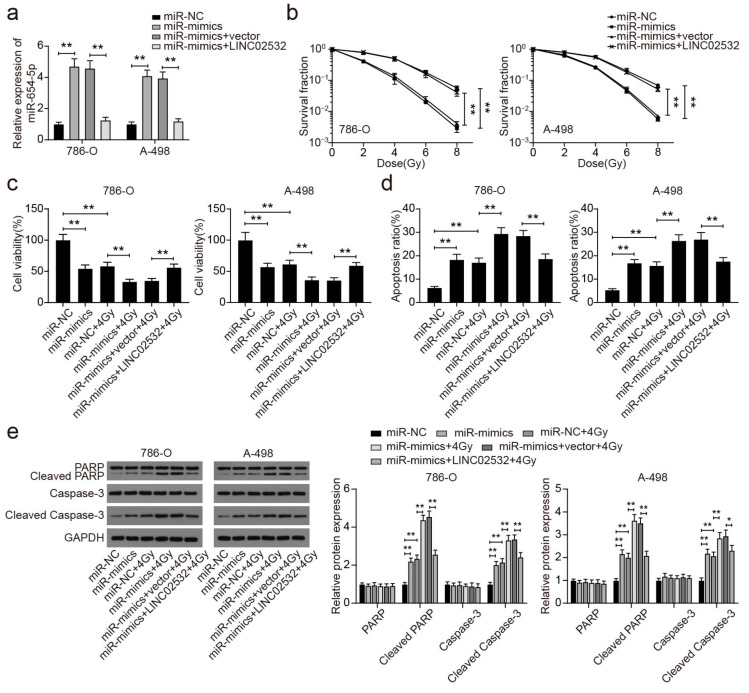
miR-654-5p overexpression diminishes the radiosensitivity of clear cell renal cell carcinoma (ccRCC) cells. (**a**) The expression of miR-654-5p in 786-O and A-498 cells transfected with miR-NC, miR-654-5p mimic, miR-654-5p + vector, or miR-654-5p + LINC02532. (**b**) Surviving cell fractions were measured in ccRCC cells with various transfection agents. (**c**) The impact of miR-654-5p on the viability of the ccRCC cells. (**d**) The impact of miR-654-5p on ccRCC cell apoptosis. (**e**) The impact of miR-654-5p on the protein expression of PARP, cleaved-PARP, Caspase-3, and cleaved-Caspase-3 in ccRCC cells. * *p* < 0.05, ** *p* < 0.01.

We further investigated the role of miR-654-5p in ccRCC cell radiosensitivity via qRT-PCR, and the results revealed that miR-654-5p expression was upregulated following transfection with miR-mimics and that this effect was restored by the upregulation of LINC02532 (Figure 5a). Further functional analyses indicated that miR-654-5p overexpression diminished surviving fractions (Figure 5b) and viability (Figure 5c), promoted cell apoptosis (Figure 5d and Figure S2), and increased the protein levels of cleaved PARP and cleaved-Caspase-3 (Figure 5e) in IR-treated 786-O and A-498 cells. In addition, the upregulation of LINC02532 reversed the effect of miR-654-5p overexpression on 786-O and A-498 cells (Figure 5b–e), suggesting that LINC02532 weakened the radiosensitivity of ccRCC cells by sponging miR-654-5p.

### 3.6. YY1 Knockdown Abolishes the Effect of LINC02532 Overexpression on Radiosensitivity in ccRCC Cells

**Figure 6:**
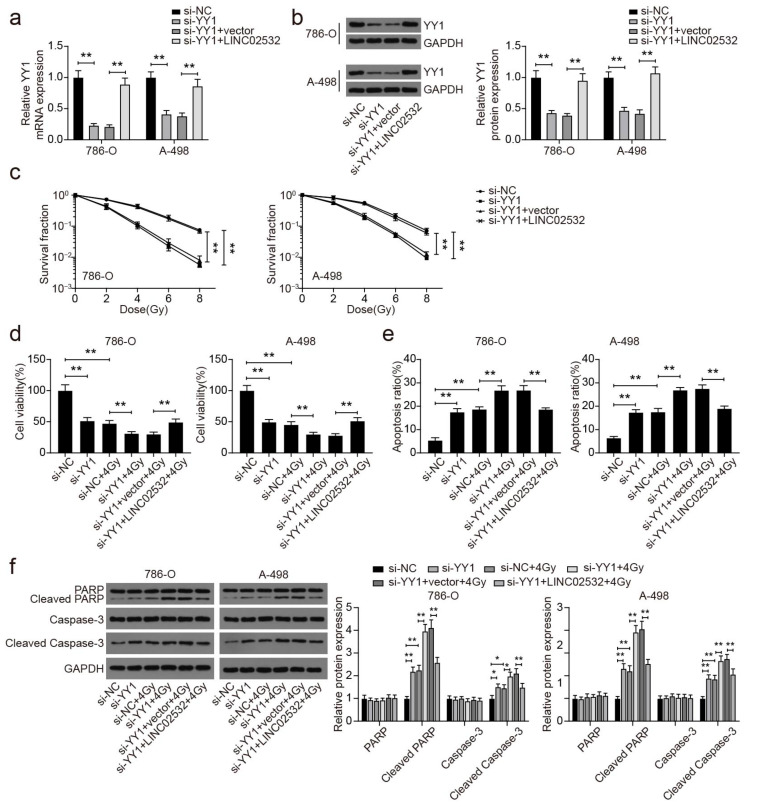
YY1 knockdown potentiates the radiosensitivity of clear cell renal cell carcinoma (ccRCC) cells. (**a**,**b**) YY1 mRNA and protein levels in 786-O and A-498 cells after transfection. (**c**) The surviving fractions of 786-O and A-498 cells after transfections. (**d**) Effect of YY1 inhibition on the viability of ccRCC cells. (**e**) Effect of YY1 inhibition on apoptosis of ccRCC cells. (**f**) The impact of miR-654-5p on the protein expression of PARP, cleaved-PARP, Caspase-3, and cleaved-Caspase-3 in ccRCC cells. * *p* < 0.05, ** *p* < 0.01.

Subsequently, we attempted to verify the role of LINC02532/YY1 in modulating the radiosensitivity of ccRCC cells. It was found that YY1 mRNA and protein expression levels were decreased by transfection with si-YY1. However, this reduction was reversed by LINC02532 overexpression (Figure 6a,b). In the cell functional assays, we observed reduced surviving cell fractions (Figure 6c) and cell viability (Figure 6d), improved cell apoptosis (Figure 6e and Figure S3), and higher levels of cleaved PARP and cleaved-Caspase-3 (Figure 6f) with YY1 knockdown in IR-treated 786-O and A-498 cells. Similar with the previous findings, these effects were reversed by the overexpression of LINC02532 (Figure 6c–f). Taken together, these results suggest that LINC02532 regulates ccRCC radiosensitivity by upregulating YY1.

### 3.7. LINC02532 Knockdown Enhances Radiosensitivity of ccRCC Cells In Vivo

**Figure 7:**
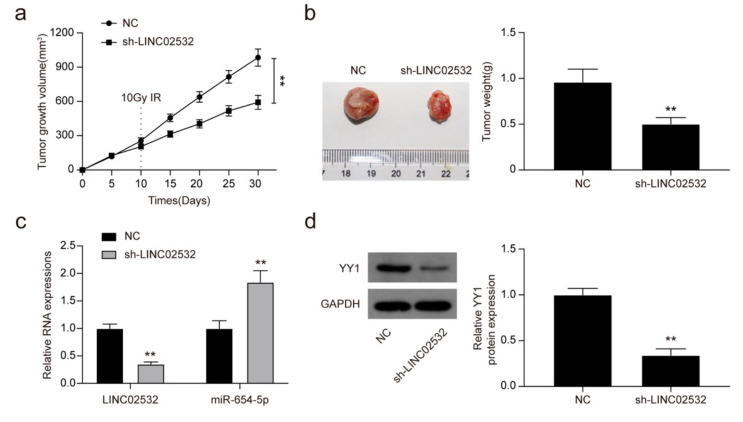
Inhibition of LINC02532 enhances the radiosensitivity of clear cell renal cell carcinoma (ccRCC) xenograft tumors. (**a**) Tumor growth was monitored every 5 d. (**b**) Tumor weight was evaluated 30 d post-injection. (**c**) Expression of LINC02532 and miR-654-5p in ccRCC xenograft tumors. (**d**) Protein expression of YY1 in ccRCC xenograft tumors. ** *p* < 0.01.

Finally, we aimed to explore whether LINC02532 regulates tumor growth under IR treatment in vivo. Subcutaneous tumors in nude mice were established using 786-O cells infected with control lentiviruses or sh-LINC02532 or lentiviruses. It was found that under IR treatment, the tumor growth was slower and the tumor weight was decreased in the sh-LINC02532 group compared with that in the control group (Figure 7a,b). Moreover, in IR-treated 786-O xenograft tumors with LINC02532 knockdown, LINC02532 and YY1 were downregulated, whereas miR-654-5p was upregulated (Figure 7c,d). Taken together, these findings suggest that the inhibition of LINC02532 enhances the radiosensitivity of ccRCC xenograft tumors through the miR-654-5p/YY1 axis.

## 4. Discussion

ccRCC, which accounts for approximately 80% of RCC cases, has the highest mortality rate among urological malignancies [45]. Radiotherapy has been widely employed as a therapeutic method for patients diagnosed with ccRCC [46,47,48]. However, the acquisition of radioresistance by the ccRCC cells affects the therapeutic effect and has become a major cause of radiotherapy failure [49]. Thus, in the present study, we aimed to investigate the molecular mechanisms related to radioresistance in ccRCC.

Rapid advances in science and technology have helped to identify potential tumor markers and new therapeutic targets, providing novel diagnostic approaches [50,51]. Moreover, whole genome sequencing helps to understand the molecular mechanisms of cancer [52,53]. Numerous studies have confirmed that lncRNAs can regulate the radiosensitivity of cancer cells, including ccRCC cells [13,54,55]. The present study focused on the lncRNA LINC02532, which is highly expressed in ccRCC cells. A previous study by Zhang et al. reported the vital role of LINC02532 in the initiation and development of gastric cancer [56]. Consistent with the previous study, we found that LINC02532 also has an oncogenic role in the development of ccRCC. Moreover, functional analysis revealed that the knockdown of LINC02532 decreased the viability of ccRCC cells. Further investigation revealed that LINC02532 expression was gradually upregulated during radiation exposure. Based on these findings, we hypothesized that LINC02532 might be associated with radioresistance in ccRCC, and subsequent experiments were performed to confirm this. As expected, LINC02532 knockdown promoted radiosensitivity in ccRCC, both in vitro and in vivo. Since the repair of DNA damage also plays a vital role in radioresistance [11], we investigated whether LINC02532 could control radioresistance by repairing DNA damage in ccRCC. The results demonstrated that LINC02532 knockdown impaired the ability of ccRCC cells to repair DNA DSBs. In summary, these data indicate that LINC02532 affected radioresistance in ccRCC by promoting DNA DSB repair in ccRCC cells. lncRNAs can be upregulated by upstream transcriptional activators. For example, FOXO1 activates DNM3OS in esophageal squamous cell carcinoma [57]. Similarly, LINC00460, which contributes to radioresistance in colorectal cancer, is activated by c-Jun [58]. YY1 is a transcriptional co-regulator that acts as a transcriptional activator of various lncRNAs [31,34,59,60]. However, whether YY1 regulates LINC02532 in ccRCC remains unclear. In this study, we found that YY1 is overexpressed in ccRCC cells. Moreover, when upregulated, YY1 bound to the LINC02532 promoter and enhanced its expression in ccRCC cells. YY1 has also been associated with the regulation of radioresistance in various cancers [36,37,61]. Consistent with previous reports, our data demonstrated that YY1 inhibition promoted the radiosensitivity of ccRCC cells. In addition, rescue experiments showed that YY1 knockdown relieved LINC02532 overexpression and weakened radiosensitivity in ccRCC cells.

The functions of lncRNAs vary and are affected by their cellular location. lncRNAs in the nucleus affect gene expression by regulating the activity of transcription factors, whereas those in the cytoplasm sponge miRNAs and regulate miRNA-targeted mRNAs at the post-transcriptional level [62,63]. In gastric cancer cells, LINC02532 is located in the cytoplasm and promotes gastric cancer cell proliferation, migration, and invasion by sponging miR-129-5p and miR-490-5p [56]. In this study, we found that LINC02532 was also mainly located in the cytoplasm of ccRCC cells. Further, through bioinformatics analysis using starBase and TargetScan datasets, LINC02532 was predicted to sponge miR-654-5p to regulate YY1, and this was subsequently verified by the luciferase reporter assays with ccRCC cells. miR-654-5p is an anti-tumor miRNA in colorectal cancer [28], osteosarcoma [29], breast cancer [27], and ovarian cancer [64]. However, its role in ccRCC has not yet been reported. Similar to previous reports, miR-654-5p in this study was upregulated in ccRCC cells, and its overexpression diminished cell viability and promoted apoptosis in ccRCC cells. Since miR-654-5p upregulation has been reported to attenuate chemoresistance in ovarian cancer [65] and non-small-cell lung cancer [66], we further investigated whether it could control radioresistance in ccRCC. The results revealed that miR-654-5p overexpression potentiated the radiosensitivity of ccRCC cells by reducing the surviving cell fraction and cell viability and increasing cell apoptosis in IR-exposed ccRCC cells. Rescue experiments further demonstrated that miR-654-5p overexpression rescued the effect of LINC02532 overexpression on the radiosensitivity of ccRCC cells.

In summary, our study demonstrated that LINC02532 knockdown potentiates the radiosensitivity of ccRCC cells in vitro and in vivo through the miR-654-5p/YY1 axis, providing insights into the role and molecular mechanism of LINC02532 in regulating ccRCC tumorigenesis and radioresistance. This implies a possible theoretical basis for the prevention of radioresistance in ccRCC. However, we also note that there were some limitations to our study. Since the molecular mechanism of lncRNAs is complicated, it is possible that LINC02532 might have other targets that exert their biological functions. Further, only 786-O and A-498 ccRCC cells were used in the present study. Hence, to confirm our findings, radioresistant cell lines should be used in future studies.

## 5. Conclusions

**Figure 8:**
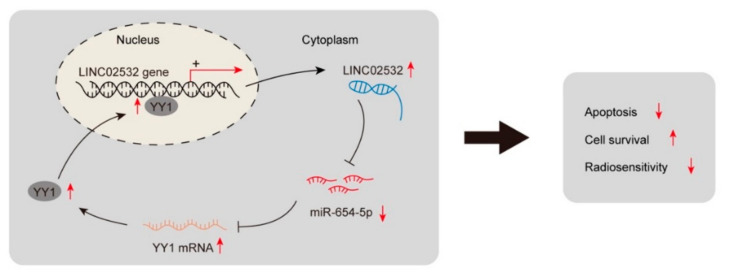
A mechanistic scheme shows that YY1-induced LINC02532 facilitates ccRCC radioresistance through the miR-654-5p/YY1 positive feedback loop.

In conclusion, our study revealed that the LINC02532/miR-654-5p/YY1 feedback loop contributes to radioresistance in ccRCC (Figure 8). This might provide new insights into overcoming radioresistance in ccRCC.

## Data Availability

The datasets used or analyzed during the current study are available from the corresponding author upon reasonable request.

## Supplementary Materials

The following are available online, Figure S1: Effect of LINC02532 inhibition on the apoptosis of clear cell renal cell carcinoma cells under IR treatment, Figure S2: The impact of miR-654-5p on clear cell renal cell carcinoma cell apoptosis, Figure S3: Effect of YY1 inhibition on apoptosis of clear cell renal cell carcinoma cells, Table S1: PCR primers used in this study, Table S2: Primers of LINC02532 promoter for YY1 occupancy, Table S3: LncRNA expressions in normal cells and ccRCC primary cells.

## Sample Availability

Samples of the compounds transfection plasmids are available from the authors.

## Abbreviations

ATCC	American Type Culture Collection
CCK-8	cell counting kit-8
ccRCC	clear cell renal cell carcinoma
ChIP	chromatin immunoprecipitation
DAPI	4′,6-diamidino-2-phenylindol
DSB	double-strand break
FBS	fetal bovine serum
FISH	fluorescence in situ hybridization
IR	ionizing radiation
lncRNA	long non-coding RNA
miRNA	microRNA
ncRNA	non-coding RNA
qRT-PCR	quantitative real-time polymerase chain reaction
RCC	renal cell carcinoma
siRNA	small interfering RNA
TCGA	The Cancer Genome Atlas
UTR	untranslated region
YY1	Yin Yang 1
