# Supplementary material for: LINC02532 Contributes to Radiosensitivity in Clear Cell Renal Cell Carcinoma through the miR-654-5p/YY1 Axis
Source: Molecules. 2021 Nov 22;26(22):7040. doi: 10.3390/molecules26227040 (PMC8625588; doi:10.3390/molecules26227040)
Supplement: Supplementary file 1 [file molecules-26-07040-s001.zip › Supplementary Figures.pdf]

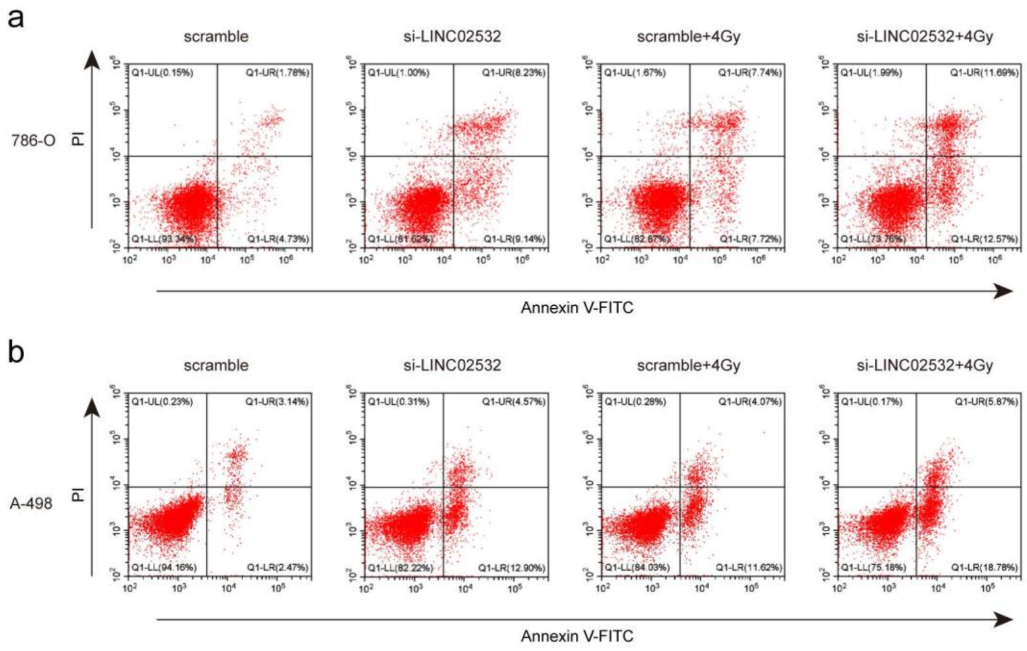

Supplementary Figure S1 Effect of LINC02532 inhibition on the apoptosis of clear cell renal cell carcinoma cells under IR treatment. (a) Representative images of cell apoptosis detected by flow cytometry in 786-O cells. (b) Representative images of cell apoptosis detected by flow cytometry in A-498 cells.

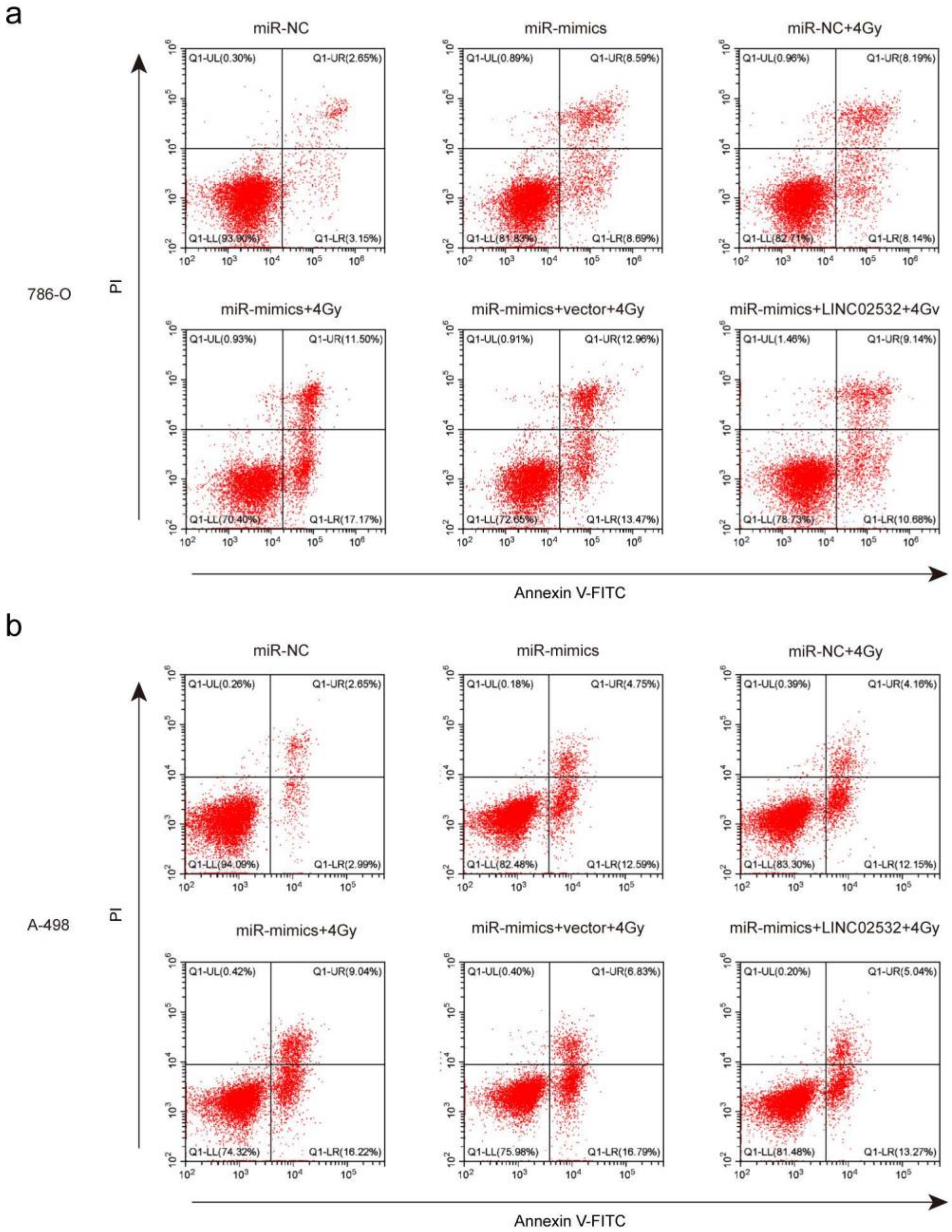

Supplementary Figure S2 The impact of miR-654-5p on clear cell renal cell carcinoma cell apoptosis. (a) Representative images of cell apoptosis detected by flow cytometry in 786-O cells. (b) Representative images of cell apoptosis detected by flow cytometry in A-498 cells.

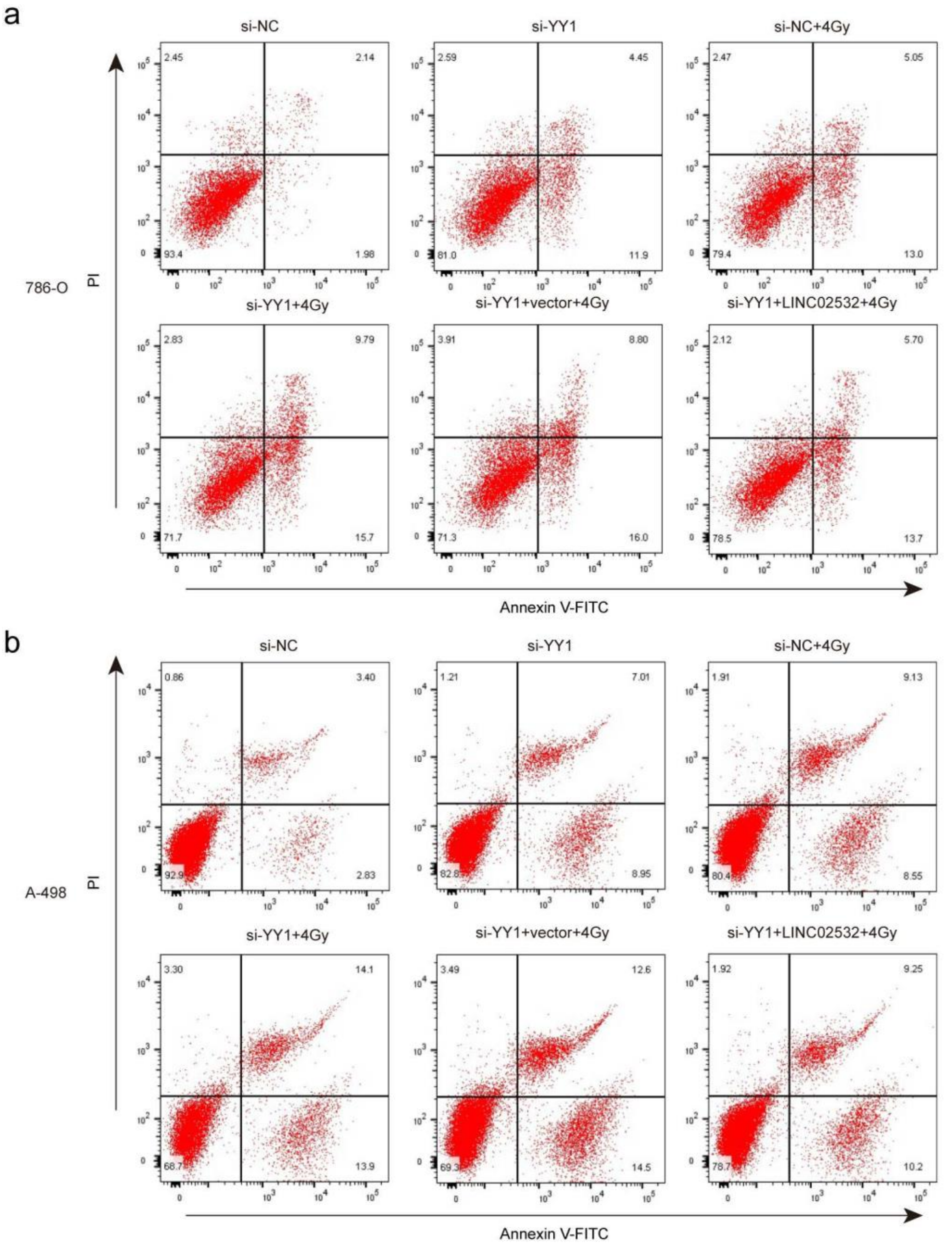

Supplementary Figure S3 Effect of YY1 inhibition on apoptosis of clear cell renal cell carcinoma cells. (a) Representative images of cell apoptosis detected by flow cytometry in 786-O cells. (b) Representative images of cell apoptosis detected by flow cytometry in A-498 cells.
