# Supplementary material for: LINC02532 Contributes to Radiosensitivity in Clear Cell Renal Cell Carcinoma through the miR-654-5p/YY1 Axis
Source: Molecules. 2021 Nov 22;26(22):7040. doi: 10.3390/molecules26227040 (PMC8625588; doi:10.3390/molecules26227040)
Supplement: Supplementary file 1 [file molecules-26-07040-s001.zip › Tables S1 and S2.pdf]

Supplementary Table S1. PCR primers used in this study.

Primers	Sequences
LINC02532(Forward)	5'-AGCCAGGACTCTTGGTAGGA-3'
LINC02532(Reverse)	5'-GATGATGCCAAGCCACCCAT-3'
miR-654-5p(Forward)	5'-TGGTGGGCCGCAGAAC-3'
miR-654-5p(Reverse)	5'-AGTGCAGGGTCCGAGGT-3'
YY1(Forward)	5'-ACGGCTTCGAGGATCAGATTC-3'
YY1(Reverse)	5'-TGACCAGCGTTTGTTCAATGT-3'
U6(Forward)	5'-CTCGCTTCGGCAGCACATATA-3'
U6(Reverse)	5'-AACGCTTCACGAATTTGCGT-3'
GAPDH(Forward)	5'-AAATCCCATCACCATCTTCC-3'
GAPDH(Reverse)	5'-TCACACCCATGACGAACA-3'

Supplementary Table S2. Primers of LINC02532 promoter for YY1 occupancy.

Primers	Sequences
LINC02532 promoter (Forward)	5'-GAGAGGGAGAGCTCAGCAG-3'
LINC02532 promoter (Reverse)	5'-TGGAACACCAAGATTTACTGGAC-3'
